# Diaqua­bis­[5-(pyrazin-2-yl-κ*N* ^1^)-3-(pyridin-3-yl)-1,2,4-triazolido-κ*N* ^1^]cadmium

**DOI:** 10.1107/S1600536811045545

**Published:** 2011-11-05

**Authors:** Jing-Jing Yang, Jun Zhao

**Affiliations:** a College of Mechanical & Material Engineering, China Three Gorges University, Yichang 443002, People’s Republic of China

## Abstract

In the title compound, [Cd(C_11_H_7_N_6_)_2_(H_2_O)_2_], the Cd^II^ cation is located on an inversion center and is coordinated by four N atoms from two 5-(pyrazin-2-yl)-3-(pyridin-3-yl)-1,2,4-triazol­ide anions and two water mol­ecules in a distorted octa­hedral geometry. The triazolide ligand is nearly planar: the central triazole ring is oriented at dihedral angles of 4.63 (13) and 8.41 (13)° with respect to the pyrazine and pyridine rings. Inter­molecular O—H⋯N hydrogen bonds link the mol­ecules into a two-dimensional supra­molecular network parallel to (001).

## Related literature

For background to metal-organic frameworks, see: Kitagawa *et al.* (2004 ▶). For 1,2,4-triazole derivatives, see: Chen *et al.* (2006 ▶); Zhang *et al.* (2005 ▶).
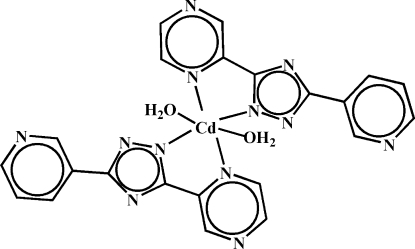


## Experimental

### 

#### Crystal data

[Cd(C_11_H_7_N_6_)_2_(H_2_O)_2_]
*M* *_r_* = 594.88
Monoclinic, 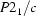

*a* = 8.640 (5) Å
*b* = 5.684 (3) Å
*c* = 23.157 (13) Å
β = 99.102 (6)°
*V* = 1122.9 (11) Å^3^
*Z* = 2
Mo *K*α radiation
μ = 1.02 mm^−1^
*T* = 296 K
0.24 × 0.21 × 0.20 mm

#### Data collection

Bruker SMART CCD diffractometer
Absorption correction: multi-scan (*SADABS*; Sheldrick, 1996 ▶) *T*_min_ = 0.782, *T*_max_ = 0.815
11387 measured reflections
2571 independent reflections
2251 reflections with *I* > 2σ(*I*)
*R*_int_ = 0.057

#### Refinement

*R*[*F* ^2^ > 2σ(*F* ^2^)] = 0.032
*wR*(*F* ^2^) = 0.081
*S* = 1.09
2571 reflections
175 parameters
3 restraints
H atoms treated by a mixture of independent and constrained refinement
Δρ_max_ = 0.38 e Å^−3^
Δρ_min_ = −0.82 e Å^−3^

### 

Data collection: *SMART* (Bruker, 2007 ▶); cell refinement: *SAINT* (Bruker, 2007 ▶); data reduction: *SAINT*; program(s) used to solve structure: *SHELXTL* (Sheldrick, 2008 ▶); program(s) used to refine structure: *SHELXTL*; molecular graphics: *SHELXTL*; software used to prepare material for publication: *SHELXTL*.

## Supplementary Material

Crystal structure: contains datablock(s) I, global. DOI: 10.1107/S1600536811045545/xu5363sup1.cif

Structure factors: contains datablock(s) I. DOI: 10.1107/S1600536811045545/xu5363Isup2.hkl

Additional supplementary materials:  crystallographic information; 3D view; checkCIF report

## Figures and Tables

**Table 1:** Selected bond lengths (Å)

Cd1—O1	2.312 (2)
Cd1—N1	2.397 (2)
Cd1—N3	2.323 (2)

**Table 2:** Hydrogen-bond geometry (Å, °)

*D*—H⋯*A*	*D*—H	H⋯*A*	*D*⋯*A*	*D*—H⋯*A*
O1—H1*A*⋯N6^i^	0.85 (2)	1.95 (2)	2.751 (3)	157 (3)
O1—H1*B*⋯N4^ii^	0.86 (2)	1.91 (2)	2.763 (3)	173 (3)

Symmetry codes: (i) 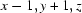; (ii) 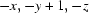.

**Fig. 1.**
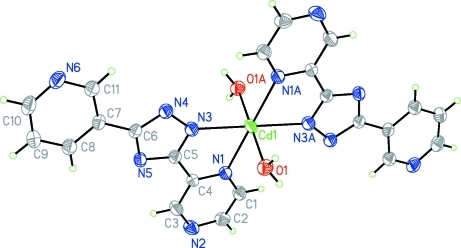
The ORTEP representation of the structure of the title compound showing the atomic numbering and 50% probability displacement ellipsoids [symmetry code: A: -x,-y + 2,-z].

**Fig. 2.**
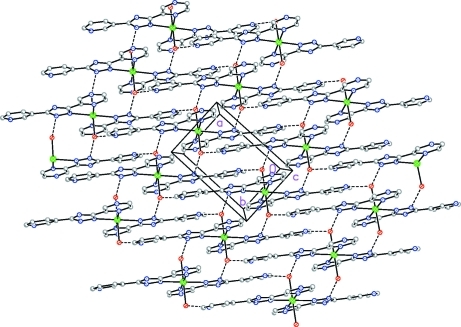
The two-dimensional network structure formed by hydrogen bonding interactions (dashed lines). H atoms are omitted for clarity.

## supplementary crystallographic information

### Comment

Much effort has been focused on the design and controlled synthesis of metal-organic frameworks or coordination polymers (Kitagawa *et al.*, 2004). 1,2,4-triazole derivatives have received considerable attention, owing to the variety of their coordination modes, and structural features (Chen *et al.*, 2006; Zhang *et al.*, 2005). During the synthesis of polymeric complexes using 2-(5-(pyridin-3-yl)-4*H*-1,2,4-triazol-3-yl)pyrazine as bridging ligand and, to our surprise, the title monomeric Cd(II) complex was obtained. The title complex, is a crstallographically centrosymmetric mononuclear complex. The Cd^II^ cation, which is located on a centre of inversion, is six-coordinated by four N atoms from two chelating 2-(5-(pyridin-3-yl)-1,2,4-triazolido-3-yl)pyrazine ligands and two water O atoms, resulting into a distored octahedral geometry (Fig. 1). In the crystal, intermolecular O–H···N hydrogen bonding interactions (Table 2) link the title complex into a two-dimensional supramolecular network (Fig. 2).

### Experimental

A mixture of 2-(5-(pyridin-3-yl)-4*H*-1,2,4-triazol-3-yl)pyrazine (0.0224 g, 0.1 mmol), Cd(CH_3_COO)_2_.2H_2_O (0.0266 g, 0.1 mmol), water (10 mL) was stired vigorously for 30 min and then sealed in a Teflon-lined stainless-steel autoclave. The autoclave was heated and maintained at 413 K for 3 d, and then cooled to room temperature at 5 K h^-1^ to obtain prism crystals suitable for X-ray analysis.

### Refinement

Water H atoms were located in a difference Fourier map and refined with O–H restraint of 0.85±0.01 Å, *U*_iso_(H) = 1.5 *U*_eq_(O). Other H-atoms were positioned geometrically and refined using a riding model with C–H = 0.93 Å, *U*_iso_(H) = 1.2 *U*_eq_(C).

### Crystal data

**Table:**

[Cd(C_11_H_7_N_6_)_2_(H_2_O)_2_]	*F*(000) = 596
*M**_r_* = 594.88	*D*_x_ = 1.759 Mg m^−^^3^
Monoclinic, *P*2_1_/*c*	Mo *K*α radiation, λ = 0.71073 Å
Hall symbol: -P 2ybc	Cell parameters from 2671 reflections
*a* = 8.640 (5) Å	θ = 2.4–27.5°
*b* = 5.684 (3) Å	µ = 1.02 mm^−^^1^
*c* = 23.157 (13) Å	*T* = 296 K
β = 99.102 (6)°	Prism, colorless
*V* = 1122.9 (11) Å^3^	0.24 × 0.21 × 0.20 mm
*Z* = 2	

### Data collection

**Table:**

Bruker SMART CCD diffractometer	2571 independent reflections
Radiation source: fine-focus sealed tube	2251 reflections with *I* > 2σ(*I*)
graphite	*R*_int_ = 0.057
φ and ω scans	θ_max_ = 27.5°, θ_min_ = 2.4°
Absorption correction: multi-scan (*SADABS*; Sheldrick, 1996)	*h* = −11→11
*T*_min_ = 0.782, *T*_max_ = 0.815	*k* = −7→7
11387 measured reflections	*l* = −30→30

### Refinement

**Table:**

Refinement on *F*^2^	Primary atom site location: structure-invariant direct methods
Least-squares matrix: full	Secondary atom site location: difference Fourier map
*R*[*F*^2^ > 2σ(*F*^2^)] = 0.032	Hydrogen site location: inferred from neighbouring sites
*wR*(*F*^2^) = 0.081	H atoms treated by a mixture of independent and constrained refinement
*S* = 1.09	*w* = 1/[σ^2^(*F*_o_^2^) + (0.0336*P*)^2^ + 0.5415*P*] where *P* = (*F*_o_^2^ + 2*F*_c_^2^)/3
2571 reflections	(Δ/σ)_max_ < 0.001
175 parameters	Δρ_max_ = 0.38 e Å^−^^3^
3 restraints	Δρ_min_ = −0.82 e Å^−^^3^

### Special details

**Table:**

Geometry. All e.s.d.'s (except the e.s.d. in the dihedral angle between two l.s. planes) are estimated using the full covariance matrix. The cell e.s.d.'s are taken into account individually in the estimation of e.s.d.'s in distances, angles and torsion angles; correlations between e.s.d.'s in cell parameters are only used when they are defined by crystal symmetry. An approximate (isotropic) treatment of cell e.s.d.'s is used for estimating e.s.d.'s involving l.s. planes.
Refinement. Refinement of *F*^2^ against ALL reflections. The weighted *R*-factor *wR* and goodness of fit *S* are based on *F*^2^, conventional *R*-factors *R* are based on *F*, with *F* set to zero for negative *F*^2^. The threshold expression of *F*^2^ > σ(*F*^2^) is used only for calculating *R*-factors(gt) *etc*. and is not relevant to the choice of reflections for refinement. *R*-factors based on *F*^2^ are statistically about twice as large as those based on *F*, and *R*- factors based on ALL data will be even larger.

### Fractional atomic coordinates and isotropic or equivalent isotropic displacement parameters (Å^2^)

**Table:**

	*x*	*y*	*z*	*U*_iso_*/*U*_eq_	
Cd1	0.0000	1.0000	0.0000	0.02997 (10)	
N1	0.0416 (2)	1.1638 (3)	0.09658 (9)	0.0272 (4)	
N2	0.0947 (3)	1.2756 (4)	0.21582 (9)	0.0365 (5)	
N3	0.1919 (2)	0.7738 (3)	0.05584 (9)	0.0295 (4)	
N4	0.2884 (2)	0.5850 (4)	0.05077 (9)	0.0306 (4)	
N5	0.3260 (2)	0.6991 (3)	0.14563 (9)	0.0290 (4)	
N6	0.6292 (3)	0.0309 (4)	0.09166 (12)	0.0395 (6)	
O1	−0.1880 (2)	0.7608 (3)	0.03044 (8)	0.0343 (4)	
H1A	−0.263 (3)	0.840 (5)	0.0406 (13)	0.051*	
H1B	−0.227 (3)	0.657 (4)	0.0055 (12)	0.051*	
C1	−0.0256 (3)	1.3500 (4)	0.11794 (11)	0.0321 (5)	
H1	−0.0932	1.4440	0.0925	0.039*	
C2	0.0024 (3)	1.4061 (5)	0.17639 (12)	0.0346 (6)	
H2	−0.0445	1.5399	0.1890	0.042*	
C3	0.1625 (3)	1.0904 (5)	0.19467 (11)	0.0340 (6)	
H3	0.2281	0.9955	0.2206	0.041*	
C4	0.1390 (3)	1.0333 (4)	0.13529 (11)	0.0257 (5)	
C5	0.2188 (3)	0.8347 (4)	0.11250 (10)	0.0266 (5)	
C6	0.3651 (3)	0.5483 (4)	0.10522 (12)	0.0272 (5)	
C7	0.4792 (3)	0.3558 (4)	0.12057 (11)	0.0288 (5)	
C8	0.5377 (3)	0.3073 (5)	0.17899 (12)	0.0343 (6)	
H8	0.5079	0.3996	0.2085	0.041*	
C9	0.6405 (3)	0.1208 (5)	0.19295 (12)	0.0379 (6)	
H9	0.6810	0.0867	0.2317	0.045*	
C10	0.6809 (3)	−0.0120 (4)	0.14809 (15)	0.0387 (7)	
H10	0.7480	−0.1390	0.1576	0.046*	
C11	0.5315 (3)	0.2144 (5)	0.07830 (12)	0.0361 (6)	
H11	0.4972	0.2485	0.0391	0.043*	

### Atomic displacement parameters (Å^2^)

**Table:**

	*U*^11^	*U*^22^	*U*^33^	*U*^12^	*U*^13^	*U*^23^
Cd1	0.03525 (16)	0.03198 (15)	0.02068 (15)	0.00690 (10)	−0.00171 (11)	−0.00100 (9)
N1	0.0299 (10)	0.0262 (9)	0.0249 (10)	0.0034 (8)	0.0026 (8)	−0.0011 (8)
N2	0.0473 (13)	0.0357 (11)	0.0264 (11)	0.0039 (10)	0.0051 (10)	−0.0055 (9)
N3	0.0328 (10)	0.0286 (10)	0.0265 (11)	0.0071 (9)	0.0029 (8)	−0.0022 (8)
N4	0.0332 (11)	0.0292 (10)	0.0290 (11)	0.0085 (9)	0.0034 (9)	−0.0033 (9)
N5	0.0289 (10)	0.0302 (10)	0.0264 (10)	0.0061 (8)	0.0001 (8)	−0.0010 (8)
N6	0.0334 (12)	0.0420 (13)	0.0434 (15)	0.0122 (10)	0.0067 (11)	−0.0048 (10)
O1	0.0345 (9)	0.0326 (9)	0.0357 (10)	0.0041 (8)	0.0055 (8)	−0.0061 (8)
C1	0.0355 (13)	0.0287 (12)	0.0318 (13)	0.0068 (10)	0.0044 (11)	0.0012 (10)
C2	0.0423 (14)	0.0292 (12)	0.0342 (14)	0.0054 (11)	0.0119 (11)	−0.0028 (11)
C3	0.0411 (14)	0.0338 (13)	0.0251 (13)	0.0057 (11)	−0.0005 (11)	−0.0005 (11)
C4	0.0249 (11)	0.0282 (11)	0.0237 (12)	0.0012 (9)	0.0029 (9)	0.0017 (9)
C5	0.0262 (11)	0.0264 (11)	0.0269 (12)	0.0027 (9)	0.0036 (9)	−0.0001 (9)
C6	0.0237 (11)	0.0295 (11)	0.0281 (13)	0.0035 (9)	0.0033 (10)	−0.0002 (10)
C7	0.0256 (11)	0.0282 (11)	0.0316 (13)	0.0043 (9)	0.0018 (10)	0.0007 (10)
C8	0.0331 (13)	0.0359 (13)	0.0330 (14)	0.0057 (11)	0.0030 (11)	−0.0010 (11)
C9	0.0355 (14)	0.0410 (14)	0.0357 (15)	0.0077 (12)	0.0011 (11)	0.0070 (12)
C10	0.0323 (13)	0.0335 (14)	0.0497 (19)	0.0104 (11)	0.0045 (13)	0.0053 (11)
C11	0.0330 (13)	0.0431 (14)	0.0315 (14)	0.0084 (11)	0.0031 (11)	−0.0025 (11)

### Geometric parameters (Å, °)

**Table:**

Cd1—O1^i^	2.312 (2)	O1—H1A	0.850 (17)
Cd1—O1	2.312 (2)	O1—H1B	0.859 (17)
Cd1—N1^i^	2.397 (2)	C1—C2	1.374 (4)
Cd1—N1	2.397 (2)	C1—H1	0.9300
Cd1—N3	2.323 (2)	C2—H2	0.9300
Cd1—N3^i^	2.323 (2)	C3—C4	1.396 (4)
N1—C1	1.339 (3)	C3—H3	0.9300
N1—C4	1.351 (3)	C4—C5	1.464 (3)
N2—C3	1.334 (3)	C6—C7	1.478 (3)
N2—C2	1.338 (3)	C7—C11	1.396 (4)
N3—C5	1.341 (3)	C7—C8	1.395 (4)
N3—N4	1.375 (3)	C8—C9	1.388 (4)
N4—C6	1.345 (3)	C8—H8	0.9300
N5—C5	1.347 (3)	C9—C10	1.373 (4)
N5—C6	1.351 (3)	C9—H9	0.9300
N6—C10	1.335 (4)	C10—H10	0.9300
N6—C11	1.347 (3)	C11—H11	0.9300
			
O1^i^—Cd1—O1	180.00 (9)	N2—C2—C1	122.4 (2)
O1^i^—Cd1—N3	91.20 (8)	N2—C2—H2	118.8
O1—Cd1—N3	88.80 (8)	C1—C2—H2	118.8
O1^i^—Cd1—N3^i^	88.80 (8)	N2—C3—C4	122.8 (2)
O1—Cd1—N3^i^	91.20 (8)	N2—C3—H3	118.6
N3—Cd1—N3^i^	180.00 (9)	C4—C3—H3	118.6
O1^i^—Cd1—N1^i^	87.30 (7)	N1—C4—C3	120.3 (2)
O1—Cd1—N1^i^	92.70 (7)	N1—C4—C5	117.6 (2)
N3—Cd1—N1^i^	107.04 (7)	C3—C4—C5	122.1 (2)
N3^i^—Cd1—N1^i^	72.96 (7)	N3—C5—N5	114.0 (2)
O1^i^—Cd1—N1	92.70 (7)	N3—C5—C4	122.2 (2)
O1—Cd1—N1	87.30 (7)	N5—C5—C4	123.8 (2)
N3—Cd1—N1	72.96 (7)	N4—C6—N5	114.3 (2)
N3^i^—Cd1—N1	107.04 (7)	N4—C6—C7	123.5 (2)
N1^i^—Cd1—N1	180.00 (4)	N5—C6—C7	122.2 (2)
C1—N1—C4	116.8 (2)	C11—C7—C8	117.3 (2)
C1—N1—Cd1	129.95 (16)	C11—C7—C6	122.3 (2)
C4—N1—Cd1	113.06 (15)	C8—C7—C6	120.4 (2)
C3—N2—C2	115.8 (2)	C9—C8—C7	119.8 (2)
C5—N3—N4	105.73 (19)	C9—C8—H8	120.1
C5—N3—Cd1	113.59 (15)	C7—C8—H8	120.1
N4—N3—Cd1	140.64 (16)	C10—C9—C8	118.3 (3)
C6—N4—N3	104.86 (19)	C10—C9—H9	120.9
C5—N5—C6	101.1 (2)	C8—C9—H9	120.9
C10—N6—C11	117.8 (2)	N6—C10—C9	123.7 (2)
Cd1—O1—H1A	112 (2)	N6—C10—H10	118.2
Cd1—O1—H1B	115 (2)	C9—C10—H10	118.2
H1A—O1—H1B	108 (2)	N6—C11—C7	123.1 (3)
N1—C1—C2	121.9 (2)	N6—C11—H11	118.5
N1—C1—H1	119.1	C7—C11—H11	118.5
C2—C1—H1	119.1		
			
O1^i^—Cd1—N1—C1	88.8 (2)	Cd1—N1—C4—C5	7.6 (3)
O1—Cd1—N1—C1	−91.2 (2)	N2—C3—C4—N1	−1.6 (4)
N3—Cd1—N1—C1	179.3 (2)	N2—C3—C4—C5	177.7 (2)
N3^i^—Cd1—N1—C1	−0.7 (2)	N4—N3—C5—N5	−0.3 (3)
N1^i^—Cd1—N1—C1	−49.9 (6)	Cd1—N3—C5—N5	177.79 (16)
O1^i^—Cd1—N1—C4	−97.06 (17)	N4—N3—C5—C4	179.0 (2)
O1—Cd1—N1—C4	82.94 (17)	Cd1—N3—C5—C4	−2.9 (3)
N3—Cd1—N1—C4	−6.62 (16)	C6—N5—C5—N3	0.3 (3)
N3^i^—Cd1—N1—C4	173.38 (16)	C6—N5—C5—C4	−179.0 (2)
N1^i^—Cd1—N1—C4	124.2 (4)	N1—C4—C5—N3	−3.4 (3)
O1^i^—Cd1—N3—C5	97.31 (17)	C3—C4—C5—N3	177.4 (2)
O1—Cd1—N3—C5	−82.69 (17)	N1—C4—C5—N5	175.8 (2)
N3^i^—Cd1—N3—C5	−54 (63)	C3—C4—C5—N5	−3.5 (4)
N1^i^—Cd1—N3—C5	−175.14 (16)	N3—N4—C6—N5	0.0 (3)
N1—Cd1—N3—C5	4.86 (16)	N3—N4—C6—C7	178.2 (2)
O1^i^—Cd1—N3—N4	−85.7 (3)	C5—N5—C6—N4	−0.2 (3)
O1—Cd1—N3—N4	94.3 (3)	C5—N5—C6—C7	−178.4 (2)
N3^i^—Cd1—N3—N4	123 (62)	N4—C6—C7—C11	8.4 (4)
N1^i^—Cd1—N3—N4	1.9 (3)	N5—C6—C7—C11	−173.5 (2)
N1—Cd1—N3—N4	−178.1 (3)	N4—C6—C7—C8	−170.8 (2)
C5—N3—N4—C6	0.1 (3)	N5—C6—C7—C8	7.2 (4)
Cd1—N3—N4—C6	−177.05 (19)	C11—C7—C8—C9	−1.5 (4)
C4—N1—C1—C2	−0.1 (4)	C6—C7—C8—C9	177.7 (2)
Cd1—N1—C1—C2	173.81 (19)	C7—C8—C9—C10	−0.4 (4)
C3—N2—C2—C1	2.1 (4)	C11—N6—C10—C9	−0.3 (4)
N1—C1—C2—N2	−1.9 (4)	C8—C9—C10—N6	1.3 (4)
C2—N2—C3—C4	−0.4 (4)	C10—N6—C11—C7	−1.8 (4)
C1—N1—C4—C3	1.8 (3)	C8—C7—C11—N6	2.7 (4)
Cd1—N1—C4—C3	−173.18 (19)	C6—C7—C11—N6	−176.5 (2)
C1—N1—C4—C5	−177.5 (2)		

Symmetry codes: (i) −*x*, −*y*+2, −*z*.

### Hydrogen-bond geometry (Å, °)

**Table:**

*D*—H···*A*	*D*—H	H···*A*	*D*···*A*	*D*—H···*A*
O1—H1A···N6^ii^	0.85 (2)	1.95 (2)	2.751 (3)	157 (3)
O1—H1B···N4^iii^	0.86 (2)	1.91 (2)	2.763 (3)	173 (3)

Symmetry codes: (ii) *x*−1, *y*+1, *z*; (iii) −*x*, −*y*+1, −*z*.
